# Supplementary figures and images for: Emergence and spread of SARS-CoV-2 variants from farmed mink to humans and back during the epidemic in Denmark, June-November 2020
Source: PLoS Pathog. 2024 Jul 1;20(7):e1012039. doi: 10.1371/journal.ppat.1012039 (PMC11244769; doi:10.1371/journal.ppat.1012039)

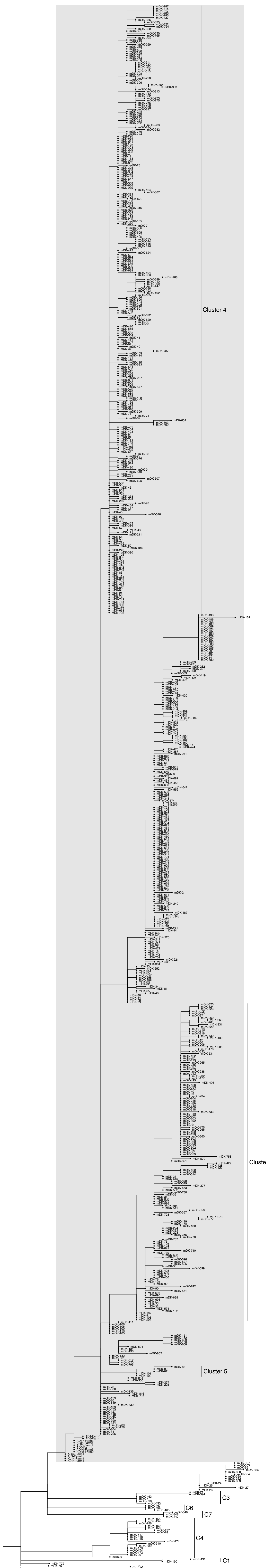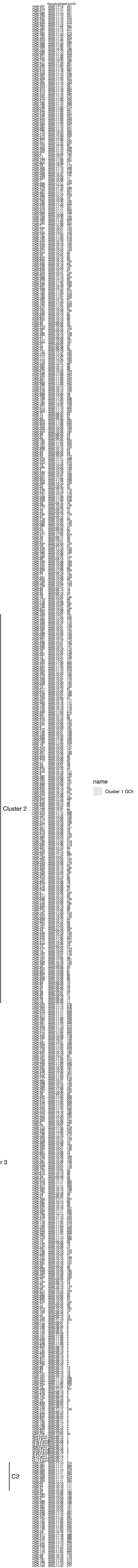

Supplement: S1 Fig — Maximum-likelihood phylogenetic tree generated using 698 mink derived virus sequences. The sampling dates and the farm identifiers are also indicated. The Clusters 2–5 (all with the Y453F substitution) are marked as are the C1-C7 variants which lack this change. (PDF) [file ppat.1012039.s003.pdf]
